# GFusion: an Effective Algorithm to Identify Fusion Genes from Cancer RNA-Seq Data

**DOI:** 10.1038/s41598-017-07070-6

**Published:** 2017-07-31

**Authors:** Jian Zhao, Qi Chen, Jing Wu, Ping Han, Xiaofeng Song

**Affiliations:** 1 Department of Biomedical Engineering, Nanjing University of Aeronautics and Astronautics, Nanjing, 210016 China; 2 Department of Gynecology and Obstetrics, The First Affiliated Hospital with Nanjing Medical University, Nanjing, 210029 China

## Abstract

Fusion gene derived from genomic rearrangement plays a key role in cancer initiation. The discovery of novel gene fusions may be of significant importance in cancer diagnosis and treatment. Meanwhile, next generation sequencing technology provide a sensitive and efficient way to identify gene fusions in genomic levels. However, there are still many challenges and limitations remaining in the existing methods which only rely on unmapped reads or discordant alignment fragments. In this work we have developed GFusion, a novel method using RNA-Seq data, to identify the fusion genes. This pipeline performs multiple alignments and strict filtering algorithm to improve sensitivity and reduce the false positive rate. GFusion successfully detected 34 from 43 previously reported fusions in four cancer datasets. We also demonstrated the effectiveness of GFusion using 24 million 76 bp paired-end reads simulation data which contains 42 artificial fusion genes, among which GFusion successfully discovered 37 fusion genes. Compared with existing methods, GFusion presented higher sensitivity and lower false positive rate. The GFusion pipeline can be accessed freely for non-commercial purposes at: https://github.com/xiaofengsong/GFusion.

## Introduction

A fusion gene is a hybrid gene formed from two different genes rejoining through chromosomal translocation, deletion or inversion^1^. Because of a close link to human cancer, fusion genes have attracted attentions of many researchers^2^. The first discovered fusion gene is BCR-ABL1^3–5^, which is formed by a translocation event involving chromosome 9 and 22 and identified as the predominant factor predisposing to chronic myelogenous leukaemia (CML). Fusion BCR-ABL1 represents one class of fusion genes which impact cancer development through encoding chimeric proteins with carcinogenic biological function. In addition, some gene fusions can lead to activation of oncogenes (for example, R-spondin fusion proteins active the oncogenic Wnt/β-catenin signaling in colon cancer) or inactivation of tumor suppressor genes (for example, LACTB2-NCOA2 disrupts NCOA2 in colorectal cancer)^6, 7^.

The existence of fusion genes in cancer such as breast, lung, colon, prostate cancers and colorectal lymphoma has been confirmed in numerous researches^8–11^. Demichelis and colleagues found that recurrent gene fusions between the androgen-regulated gene TMPRSS2 and the ETS (E26 transformation-specific) family genes ERG, ETV1 or ETV4 are expressed in most prostate cancers^12^. Tomlins and colleagues found that ERG or ETV1 was markedly overexpressed in 57% prostate cancer cases, whereas overexpression was never observed across benign prostate tissue samples^13^. Singh and colleagues found that some GBMs (glioblastoma multiforme) involve FGFR-TACC fusion and the fusion protein displays oncogenic activity when introduced into astrocytes in the mouse brain^14^. The discovery of the EML4-ALK fusion in non-small-cell lung cancer, SYT-SSX4 fusion in synovial sarcoma and many other fusion events in cancers indicated that gene fusions are widespread existed in tumor types^15–17^.

The fusion genes detections were traditionally relied on the FISH (fluorescence *in situ* hybridization) or RT-PCR techniques. With the development of next generation sequencing, many fusion genes have been identified based on RNA-seq data^18–20^. Using the RNA-seq data, several computational tools have been developed to identify fusion genes, such as FusionSeq, FusionMap, Tophat-fusion, PRADA, SOAPfuse, FusionCatcher, JAFFA, ChimPipe^21–28^. FusionSeq is a novel approach which can detect candidate fusion transcripts by analyzing paired-end RNA-Seq data. It firstly clusters a large number of short ‘tiles’ from exon sequences from discordant read pairs and then constructs a ‘fusion junction library’ which is used to realign the potential fusion reads. Thus the running cost of FusionSeq is much higher in terms of running time and CPU usage because the function junction library is normally quite large. Both of FusionMap and Tophat-Fusion can apply on the single-end or paired-end read using similar strategy that is to split reads into shorter segments and select segments aligning against different genes. FusionMap then creates a pseudo fusion transcript library based on spanning fusion boundaries reads, and remaps full-length reads to this pseudo reference, while TopHat-Fusion uses a series of post-processing routines to filter out false fusions. To detect fusion genes, the current pipelines heavily depend on individual unmapped reads which harbor the fusion boundaries or discordant paired-end reads, in which each reads align against different genes, leading to neglecting the mate reads of unmapped reads or reads that span fusion boundaries. Thus, the results contain lots of spurious fusion genes. PRADA is comprehensive software platform which can solve gene expression levels, quality metrics, detection fusion transcripts, and so on. SOAPfuse employs a series of filters to nominate high-confidence fusion transcripts from the library of fusion events idenfied by an improved partial exhaustion algorithm. FusionCatcher searches for novel/known somatic fusion genes, translocations and chimeras in RNA-seq data (paired-end or single-end reads). *JAFFA* detects fusions with different read-lengths, and it uses de novo assembly or raw reads directly to align to a reference transcriptome, rather than the genome. ChimPipe combines discordant paired-end reads and split-reads to detect any kind of chimeras. Liu *et al*. comprehensively evaluated 15 popular fusion transcript detection algorithms on three synthetic data sets and three real data sets respectively, and found that no single method dominantly performed the best for all data^29^. Shailesh *et al*. and collegues compared the performance of 12 well-known fusion detection pipelines^30^, and suggested that these 12 fusion detection tools have different false positive rate, and none of the tools are inclusive.

To improve the sensitivity and specificity of fusion detection, and avoid the above limitations, we present a novel pipeline named GFusion, a powerful and efficient fusions detection method for both paired-end and single-end RNA-seq data, to predict fusion genes by comprehensive analyzing alignment split reads and mate reads. GFusion firstly splits unmapped reads into three segments and aligns the first and last segments against reference genome using read aligner Bowtie^31^. Potential fusion boundaries are confirmed by the split reads in which the segments are from different genes. Finally, GFusion filters out false fusions through a series of filtering steps including analyzing the mate reads of split reads for paired-end read, detecting spanning fragments and constructing fusion reference and realigning fusion fragments (fusion reads for single-end data). We have demonstrated the effectiveness of the GFusion on a normal breast RNA-Seq dataset as the control group and four cancer RNA-Seq datasets involving both paired-end and single-end read data. As a result, GFusion successfully detected 34 from 43 previously reported fusions in four cancer datasets. Further results illustrated that GFusion performed higher sensitivity and have lower false positive rate by comparing with other existing fusion detection pipelines.

## Methods

**Figure 1:**
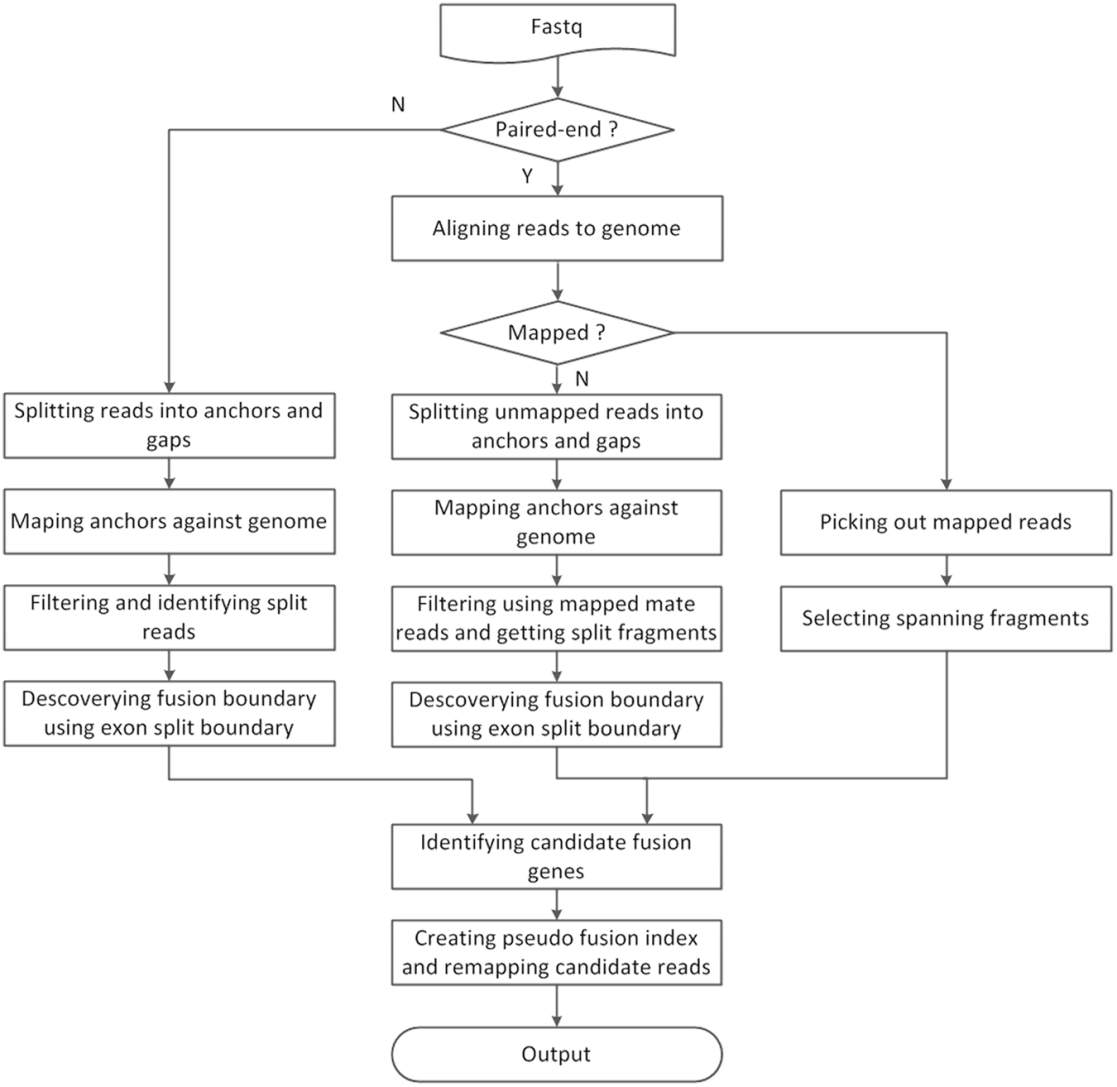
The pipeline of GFusion. GFusion employed multiple alignments and a series of post-alignment strict filtering routines for fusion gene detection.

GFusion is a Perl based software designed to identify fusion genes from single-end or paired-end RNA-Seq read data, which are aligned against the reference genome to find out the genomic locations, followed by integrated filtering steps to determine the best gene fusion candidates (Fig. 1). GFuison involves several steps to eliminate false candidate fusions that are caused by misalignment, random pairing of transcript fragments or artificial errors. We identify fusion gene candidates from selected fragments harboring the fusion boundaries in their sequenced reads or insert segments. In the output file, the GFusion reports a list of information about the fusion result including detected fusion genes, genomic locations of breakpoints, fusion transcript model, as well as the number of supporting split fragments and spanning fragments (split reads for single-end data), that characterize fusion genes comprehensively at transcript expression level.

**Figure 2:**
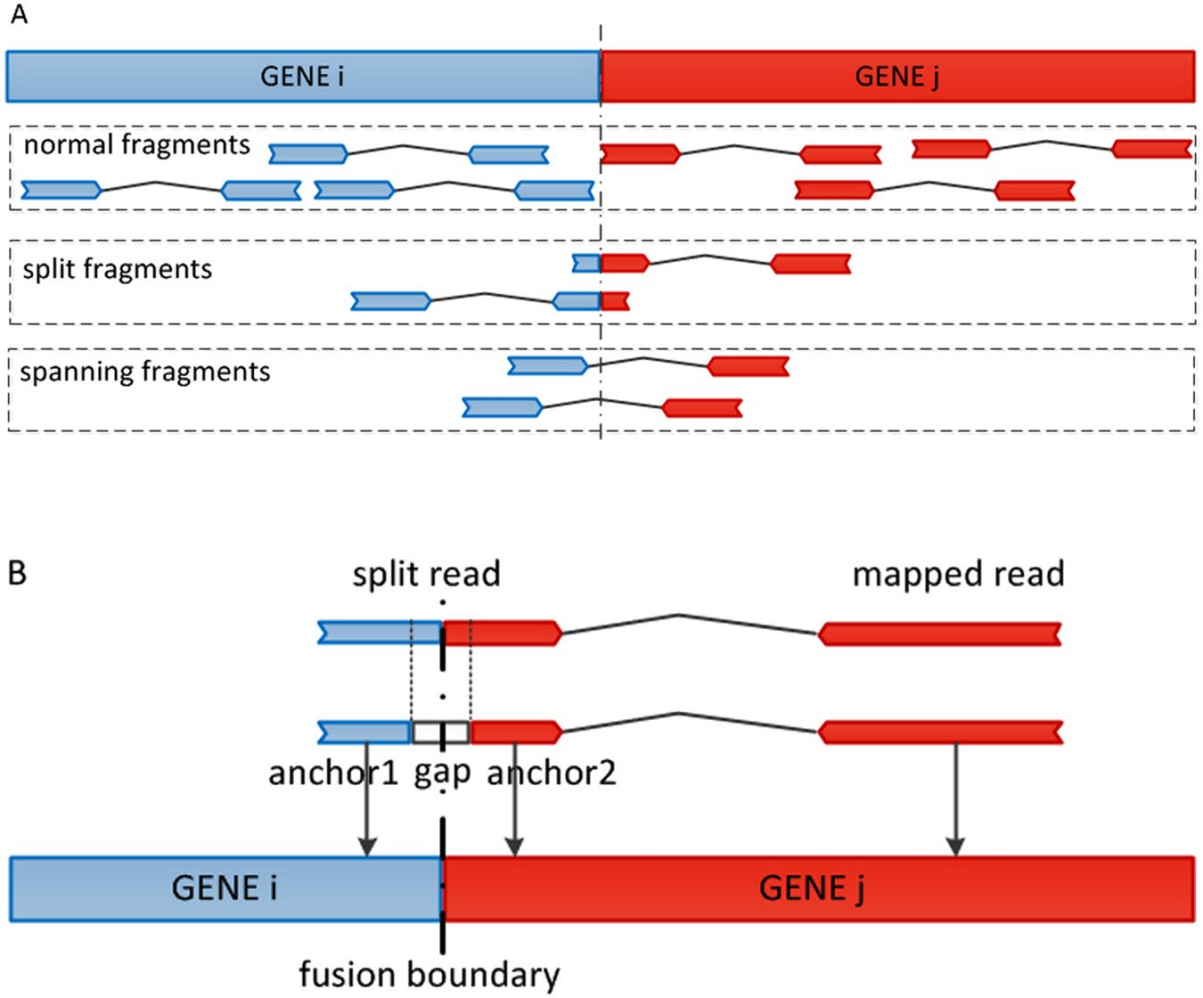
The supporting fragment or reads illustration for fusion gene in GFusion. (**A**) The normal fragment was defined as one whose read and mate are all mapped to same gene, the split fragment was defined as one that one read of fragment harbors fusion boundary and its mate was mapped to the one gene of fusion, and spanning fragments were defined as one that one read or mate of the fragment was mapped to two different genes. (**B**) The split read harbors a fusion boundary, and its mate read can be aligned to the gene of fusion.

In this work, a fusion boundary was defined as the precise genomic location as the breakpoint of the fusion genes. Two segment sequences from different genes are combined at a fusion boundary to be a fusion transcript. We defined a split read as the sequenced read which harbors a fusion boundary in the read itself, while a split fragment is the pair-end read where one read is the split read and the mate read has a special alignment related to the split read. In addition, we defined a spanning fragment as a paired-end read that harbors a fusion boundary in the insert segment with two reads being aligned to different genes (Fig. 2). The following analysis show the main steps of GFusion fusion detection procedure on paired-end RNA-Seq fastq datasets, some filters are omitted for single-end fastq datasets.

### Initial alignment

GFusion detect fusion genes based on alignment locations of reads in the genome. To determine the precise genomic location of short sequences, GFusion involves multiple uses of basic aligners. The initial step of the process is to align all the reads to the reference genome (hg19 version in this work). Because of the presence of introns in the genome, we used Tophat^32^, a fast and efficient splice aligner, for initial alignment. Tophat uses Bowite to identify the reads that can be mapped to the genome as exon reads or known exon junction spanning reads through aligning a read in small pieces to a chromosome, producing Sequence Alignment/Map (SAM)^33^ format output file and comprising every reads either mapped or unmapped against the reference genome. Some of the unmapped reads and poor quality mapping reads may be due to RNA extraction errors, sequencing bases bias and aligner algorithm defects. We discard poor quality reads by setting the mapping quality score to be higher than 30.

### Creation of anchors

The next step of the GFusion is to split unmapped reads into three shorter segments, while its mate reads should be aligned to genome with high mapping quality. The first and last segments are defined as anchors_1 and anchor_2 separately with 20 bp or longer length (0.4 by default but no greater than 0.5 of the length of reads). Middle segment is defined to be a gap and the gap length equals read length minus anchor length. For example, a 50 bp read will be split into three segments: 20 bp anchors_1, 10 bp gap, and 20 bp anchors_2. The two anchors and a gap form a special pseudo paired-read reads that the anchors are the paired-end reads and gap is the insert segment. What’s more, query name and the sequence quality values of the anchors are inherited from the parental reads with corresponding symbols to distinguish each anchor. GFusion identifies the potential split reads based on the necessary conditions that fusion boundaries are located in the gaps and the anchors are originated from different genes. In order to confirm the original genes of anchors, the split segments should be aligned against reference genome, shorter length of anchors may lead to multiple alignments, whereas the longer length of anchors makes it hard to detect the fusion boundary from narrow gaps^34^. Therefore, a best length of anchors is suggested to be 0.4 of read length.

### Aligning and locating anchors

The third step is to align the anchors independently to the reference genome by using Bowtie, an ultrafast, memory-efficient aligner for aligning un-spliced short reads to the genome. GFusion picked out the pair anchors, both of which are originated from same parental reads with common identifiers and can be aligned to genome with high mapping quality as described in the above. Then, GFusion attempts to select potential split reads with two anchors uniquely aligning to different genes and discards normal reads with the anchor pairs both matching the same genes. The paired-end reads, where the anchor_2 of potential split reads and mate reads locate in the same gene, will be considered as potential split fragment because fusion boundaries cut the paired-end reads into two segments from different genes: one consists of mate reads, insert segment, anchor_2 and the other is anchor_1. The most powerful advantage of GFusion is that, unlike traditional fusion gene detection models such as Tophat-Fusion, FusionMap and other pipelines relying on unmapped split reads or discordant alignments, it comprehensively considers the mate reads and split reads alignment results in an efficient way to make the false positive rate far lower.

### Locating fusion boundaries

To locate fusion boundaries, we proposed an evaluation criterion based on the human gene annotation (GTF file) and potential split reads which harbor the fusion boundaries in the gaps. Potential split reads with alignments where two anchors locate near the approximate exon boundaries is selected and exon junction boundaries are considered to be approximate fusion boundaries. Thus, the distance between fusion boundary and anchor alignment coordination should be less than gap length. We defined *Cor* as an anchor alignment position (the first alignment base coordination) in reference genome. The anchors aligning to forward reference are defined as *strand*+, while anchors aligning to reverse-complement reference are defined as *strand*−. We also defined *G*1 as the start coordinate of an exon, and *G*2 as the end coordinate of an exon. The abbreviation for anchor length and gap length is expected to be *al* and *gl* separately. In addition, we added a parameter (default: *p* = 3), which is defined as the maximum allowing distance between anchor boundary site and its mapped exon boundary site. The most probable range of anchor location is restricted as follows:

1 $$\{\begin{array}{ll}-p < Cor-{G}_{1} < gl+p\,, & if\,ancho{r}_{1}strand-or\,ancho{r}_{2}strand+\\ al-p < {G}_{2}-Cor < al+gl+p, & if\,ancho{r}_{1}strand+or\,ancho{r}_{2}strand-\end{array}$$

### Confirming fusion models

We defined four fusion models to denote the fusion gene strand direction based on supporting split fragment aligning orientations and gene transcribing orientations. *f f* is one fusion model that upstream gene and downstream genes are both forward transcribed and splicing in the forward orientation. Similarly fused genes both having reversed strand orientations are defined as *rr* model. Besides, *fr* is another fusion model for the fusion gene which is generated by upstream gene with forward orientation and downstream gene with reversed orientation, while fusion model *rf* confronts *fr* that fusion gene with *rf* model concatenates the reversed strand sequence of upstream gene to the forward strand sequence of downstream gene.

### Aggregating candidate fusions

For paired-end RNA-seq data, GFusion identifies candidate fusions based on the number of split fragments and spanning fragments as an evidence of gene fusion events. Spanning fragments are special discordant pair alignments harboring fusion boundaries in the insert segments with each read aligning to the different genes. To search spanning fragments, firstly we selected the aligned reads with high mapping quality scores from initial alignment results. If the reads with common query name hit different gene exons on the side of potential fusion boundaries, the read pairs are stored as potential spanning fragments. By default, GFusion only reports fusion candidates supported by at least one split fragment and one spanning fragment to remove false positive fusions.

### Constructing fusion index and realigning

After identifying the potential fusions, we constructed the bowtie reference index using the potential fusion sequences and realign all the supporting fragments against the index for further filtering. This is the last filtering step to eliminate false positives. All sequences of fusion genes on the each side of fusion boundaries are collected and concatenated to produce the pseudo fusion reference indexes. For example, when the fusion model is *fr*, GFusion extracts upstream sequence from the first base to the fusion boundary base of upstream gene and extracts downstream sequence from the fusion boundary base to the last base of downstream gene using reference genome sequence file (hg19.fa). While the fusion model is *rf*, GFusion extracts upstream sequence from the fusion boundary base to the stopping base of downstream gene with reverse-complemented transforming and extracts downstream sequence from the fusion boundary base to the stop base of upstream gene. The upstream and downstream sequences are concatenated together to produce potential fusion reference sequences at fusion boundaries. Using fusion reference sequences, GFusion is able to build fusion bowtie index for supporting fragments realigning by Bowtie. Every candidate split fragments and spanning fragments are realigned against constructing index to recount the number of supporting fragment. As described in Step6, GFusion finally confirms fusion genes with fusion boundaries, fusion transcript model supported by at least one split fragment and one spanning fragment.

### Tools selected for comparison

The fusion detection tools that we chose to benchmark together with GFusion were the following: Tophat-Fusion, FusionMap, JAFFA, and ChimPipe. Tophat-Fusion and FusionMap were chosen because they were extensively used by the community. In addition, they were also commonly used to conduct the comparison with other subsequent fusion detection tools. JAFFA and ChimPipe were chosen because they were two latest tools for fusion discovery and showed a better performance than other state-of-the-art tools. Therefore, these four tools were finally selected for comparison.

### Tophat-Fusion and FusionMap parameters

In the Tophat-Fusion, the fusion-min-dist means that for intrachromosomal fusions, Tophat-Fusion tries to find fusions separated by at least this distance, and the num-fusion-reads means that fusions with at least this many supporting reads will be reported. In the FusionMap, α denotes the minimum end length of a seed read, β denotes the maximum hits of a read end, G denotes the non-canonical splice pattern penalty, MinimalHit denotes the minimal distinct fusion reads, and MinimalFusionSpan denotes the minimal distance (bp) between two fusion break points.

## Results

We tested GFusion on three types of RNA-Seq dataset: (1) paired-end RNA-Seq datasets: three breast cancer cell lines BT474, SKBR3, MCF7 and a normal breast cell line described by Edgren *et al*. and can be available from the NCBI Sequence Read Archive [SRA:SRP003186]^35^; (2) single-end RNA-Seq datasets: the K562 CML cell line, containing 14 million 76 bp SE reads from work by Levin *et al*.^36^; (3) simulated datasets: consisting of approximately 24 million 76 bp paired-end reads and artificial fusion reads. We mapped all reads and short fragments to the human genome (hg19) with TopHat and Bowtie, and identified the genes involved in each fusion using the human gene annotations.

### Testing on Paired-end Datasets

In three cancer breast cell lines, 37 fusion genes have been discovered in the previously research^35, 37^. GFusion found 34 fusion candidates containing 28 previously reported fusions showed in Table 1 (see details in Table S2). We also ran Tophat-Fusion, FusionMap, JAFFA, and ChimPipe on the same datasets to compare performance with GFusion. Tophat-Fusion found 66 fusion candidates containing 30 known fusion events and FusionMap reported 26 candidate fusion genes, 16 of which were previously known fusion genes. JAFFA found 37 fusion candidates containing 21 known fusion events, while ChimPipe reported 38 candidate fusion genes including 30 previously known fusion genes. It can be seen that Tophat-Fusion and ChimPipe achieves the highest sensitivity (81.08%) following by GFusion (75.67%). For the precision, GFusion has the highest value (82.35%) following by ChimPipe (78.94%), while the Tophat-Fusion has the lowest value (46.45%).

**Table 1:** The number of known fusion genes and novel fusion genes reported by GFusion, Tophat-Fusion, FusionMap, JAFFA, and ChimPipe.

Sample		GFusion	Tophat-Fusion	FusionMap	JAFFA	ChimPipe
MCF7 (PreKF:6)	KF	4	4	3	4	5
	NF	2	7	3	5	3
BT-474 (PreKF:21)	KF	17	19	9	12	18
	NF	1	24	3	6	4
SK-BR-3 (PreKF:10)	KF	7	8	4	5	7
	NF	3	5	4	5	1
Normal	NF	1	4	1	3	3

KF is the number of known fusion genes reported by program. PreKF is the number of all known fusion genes in the previously reports. NF is the number of novel fusion genes reported by program.

**Figure 3:**
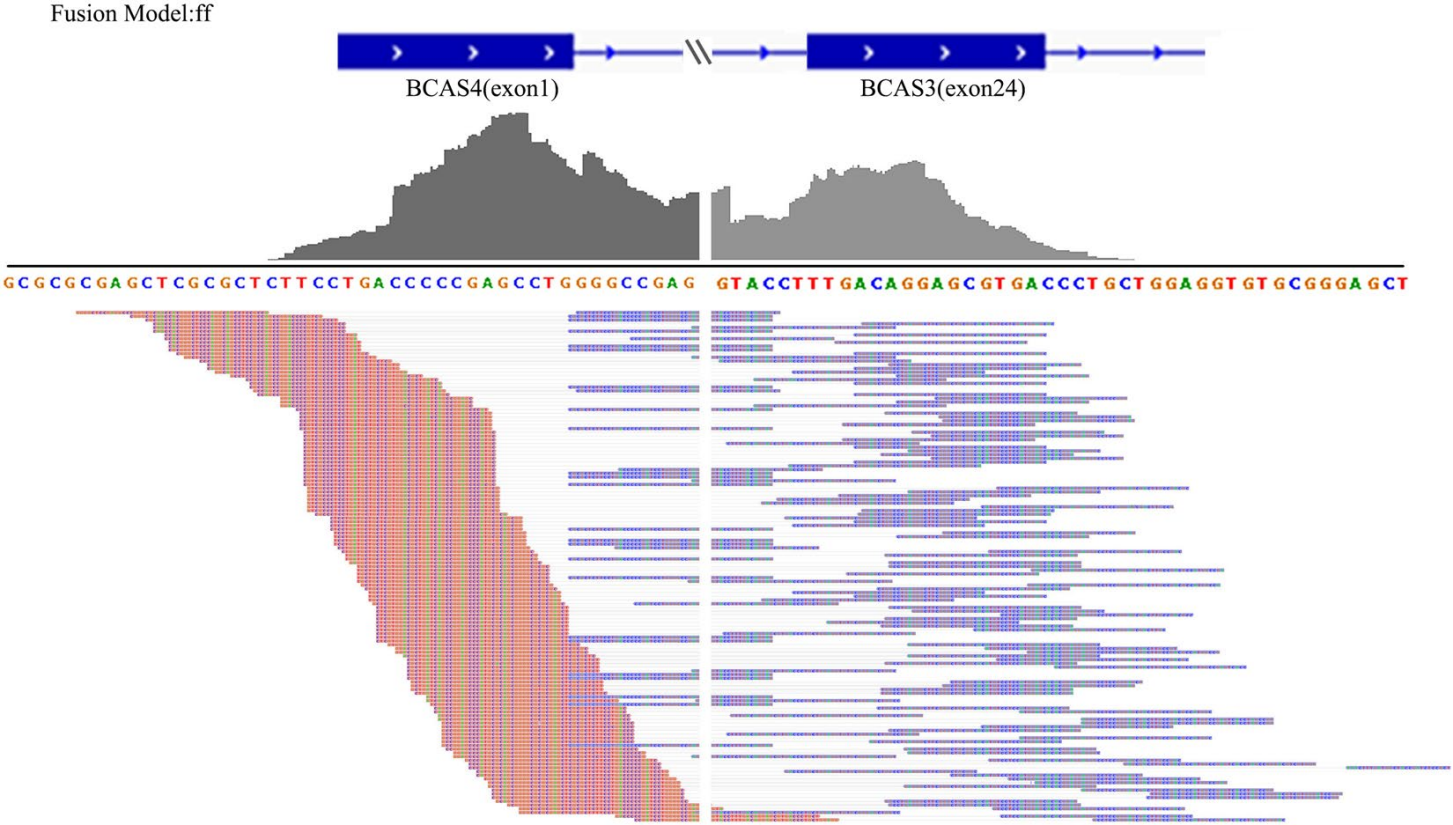
Supporting fragments of fusion gene BCAS4-BCAS3. GFusion identified the fusion supporting fragments clearly spanning the fusion boundary between the BCAS4 and BCAS3 genes on chromosomes 20 and 17 separately.

For the MCF7 cell lines, ChimPipe found 5 known fusion genes following by the 4 known fusions respectively detected by GFusion, Tophat-Fusion, and JAFFA. FusionMap only reported 3 known fusion transcripts (BCAS4-BCAS3, ARFGEF2-SULF2, RPS6KB1-VMP1), which were also found by other four tools. In addition, GFusion has the highest precision as it only reported two novel fusions (Table S2), while Tophat-Fusion reported other seven novel fusion genes so it’s precision is the lowest. In the two novel fusion genes detected by GFusion, the novel fusion (PAPOLA-AK7) was also found by other four programs except the ChimPipe. Figure 3 illustrates BCAS4-BCAS3 fusion supporting fragments identified by GFusion. The facts that fusion reads spanned the fusion boundary between the BCAS4 and BCAS3 genes on chromosomes 20 and 17 proved that BCAS4 and BCAS3 gene fused and expressed in the MCF7 breast cancer cell line.

**Figure 4:**
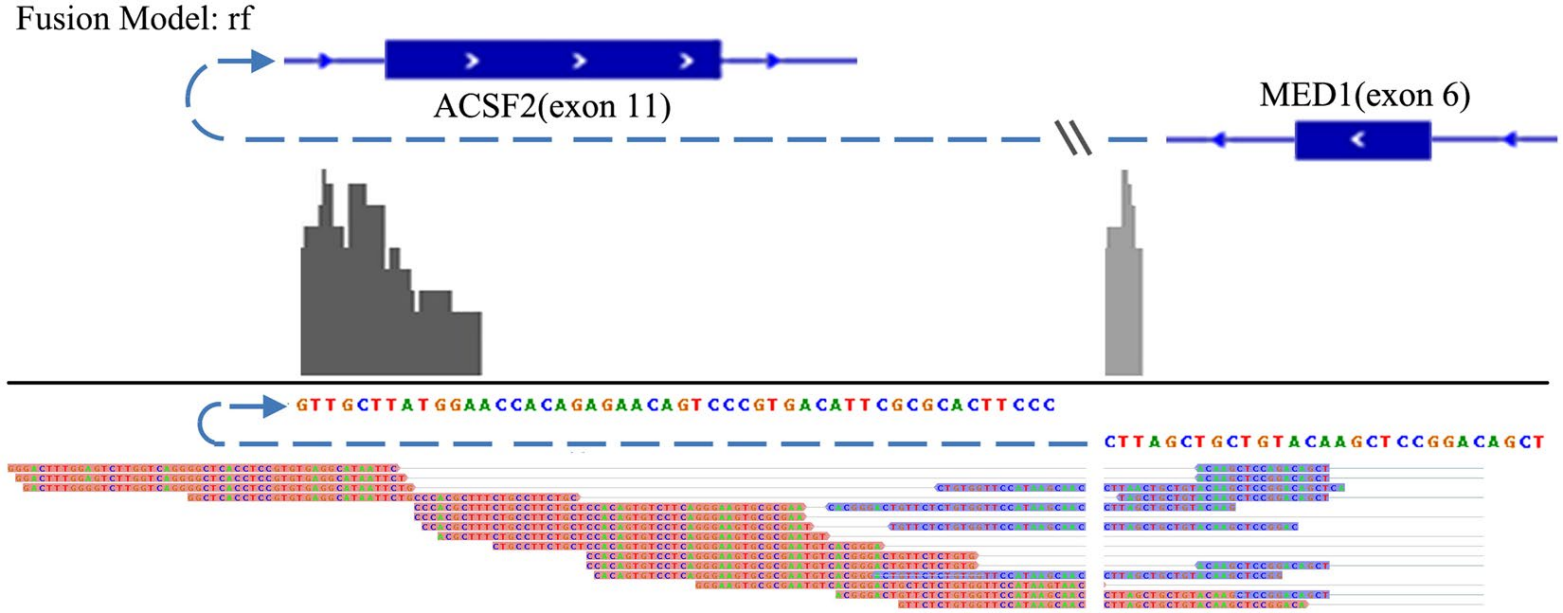
A novel fusion gene MED1-ACSF2 detected by GFusion. Six split fragments and nine spanning fragments span the fusion boundary of the two genes. The upstream fusion gene MED1 is reversed transcribed, while downstream fusion gene ACSF2 is forward transcribed.

For the BT474 cell line, GFusion detected the 17 fusions out of 21 previously reported fusion transcripts, and predicted only one novel fusion transcript (Table S2). FusionMap found 9 reported fusion genes and predicted 3 novel fusion transcripts. Algthough TopHat-Fusion found 19 known fusions, it predicted 24 novel fusions. JAFFA detected 12 known and 6 novel fusions, while ChimPipe reported 18 known and 4 novel fusions. The above results shows GFusion achieves the highest precision, and its sensitivity differs by only about 1% from that of Tophat-Fusion. Furthermore, GFusion missed one known fusion (LAMP1-MCF2L) because this fusion was supported by only two low mapping quality split fragments and it was filtered out in our initial filtering step. Figure 4 shows MED1-ACSF2 fusion supporting fragments identified by GFusion, these reads clearly span the intra-chromosomal (chromosome 17) fusion boundary of the two genes. The upstream fusion gene MED1 is reversed transcribed, while downstream fusion gene ACSF2 is forward transcribed.

**Figure 5:**
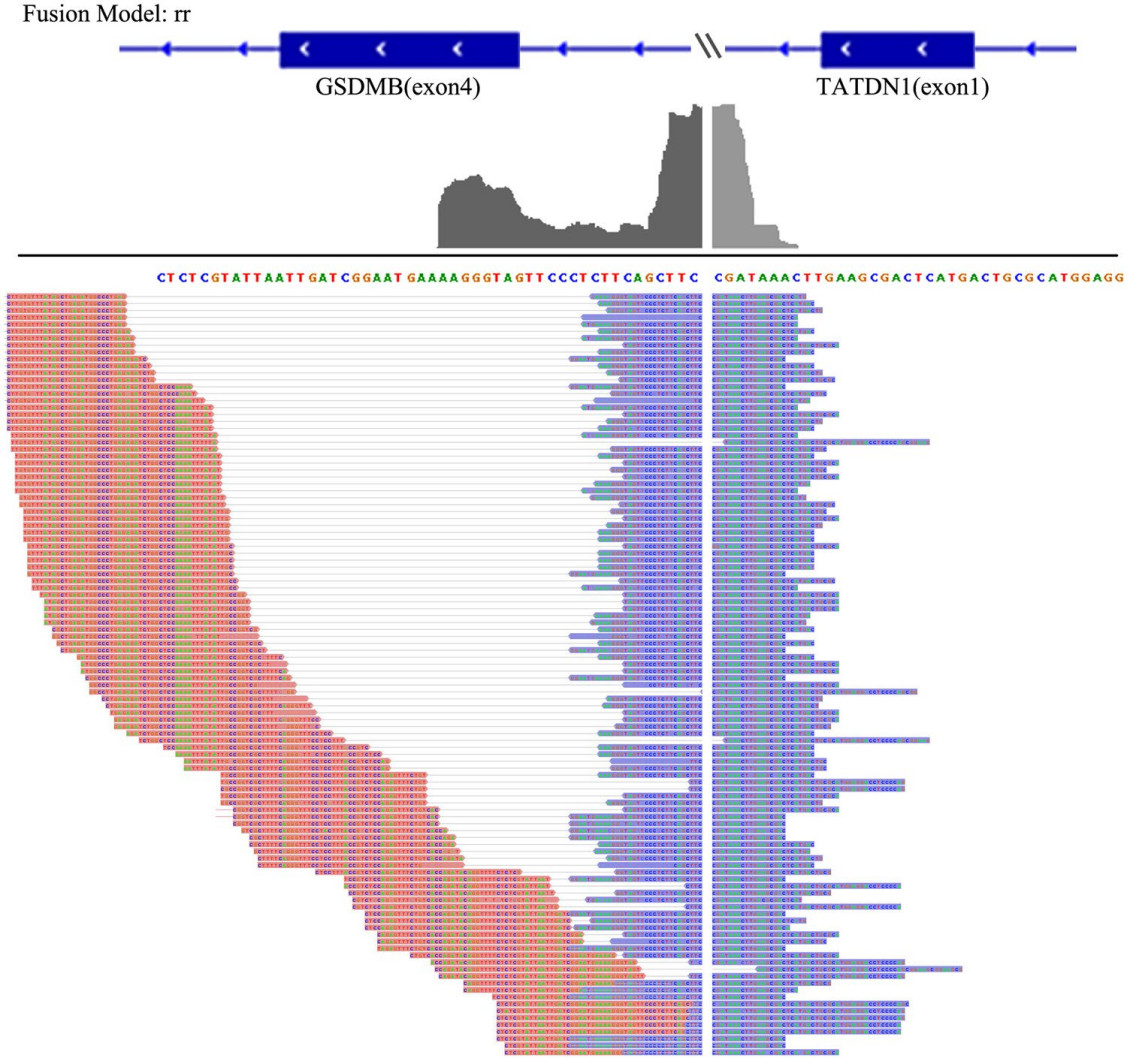
One isoform of fusion gene TATDN1-GSDMB detected by GFusion.

In the SKBR3 breast cancer cell line, GFusion, Tophat-Fusion and ChimPipe all found the same seven fusions out of ten previously reported fusion genes and predicted three (Table S2), five and one novel fusions respectively. While FusionMap missed six known fusions and found just four previously reported fusion transcripts and predicted four novel fusions. JAFFA reported five known fusions and five novel fusions. Among the fusion genes missed by GFusion, one known fusion, CSE1L-ENSG00000236127, was not found because ENSG00000236127 annotation has been removed from the recent Ensembl database. NFS1-PREX1 was filtered out because fusion supporting fragments had low mapping quality. However in the previously researches^35^, the evidences of fusion NFS1-PREX1 was not enough, as only a short segment of NFS1 was included in the fusion. Figure 5 shows the fusion supporting fragments for one of four fusion isoforms of TATDN1-GSDMB with strong alignment evidence that found by GFusion in SKBR3 cells. The upstream fusion gene TATDN1 and downstream fusion gene GSDMB are both reversed transcribed.

In fusion genes detection, the biggest challenge is to reduce the huge number of false positives resulted from error of sequencing and alignment. In order to decrease the number of false fusions, we have designed a series of strict filtering algorithms during fusion genes detection process. In order to estimate the false positive rate of three pipelines, we ran them on RNA-Seq datasets of normal breast cell line. GFusion and FusionMap both predicted just one novel fusion transcript. JAFFA and ChimPipe both predicted three novel fusion transcripts, and Tophat-Fusion predicted 4 novel fusions. These results indicate that GFusion had far fewer false positives than Tophat-Fusion, JAFFA, and ChimPipe.

In addition, it should be noted that the running time of GFusion was about 30 minutes through × 64 eight-core computer and six running threads and the majority of running time was spent on the read aligning. Using the same computer and threads, Tophat-fusion took an hour and 40 minutes, ChimPipe used an hour and 34 minutes, and FusionMap was the fast one for fusion analysis based on the fast alignment program GSNAP. JAFFA took five hours and 13 minutes with 12 threads.

In conclusion, GFusion can achieve high sensitivity and precision, and should be a reliable tool for fusion gene detection because of a series of filtering steps. Unlike Tophat-Fusion and FusionMap as well as other fusion detection tools, one of powerful features in GFusion is that it focuses more on the mapped mate reads and split reads.

### Testing on Single-end Dataset

Since recent advances in NGS platforms has resulted in vast increases in coverage and read length, gene fusion boundaries are now adequately represented by single-end reads. Fusion detection using single-end reads may be more powerful than the paired-end reads when the discordant read pairs or the short insert segment is used in detection algorithm.

**Figure 6:**
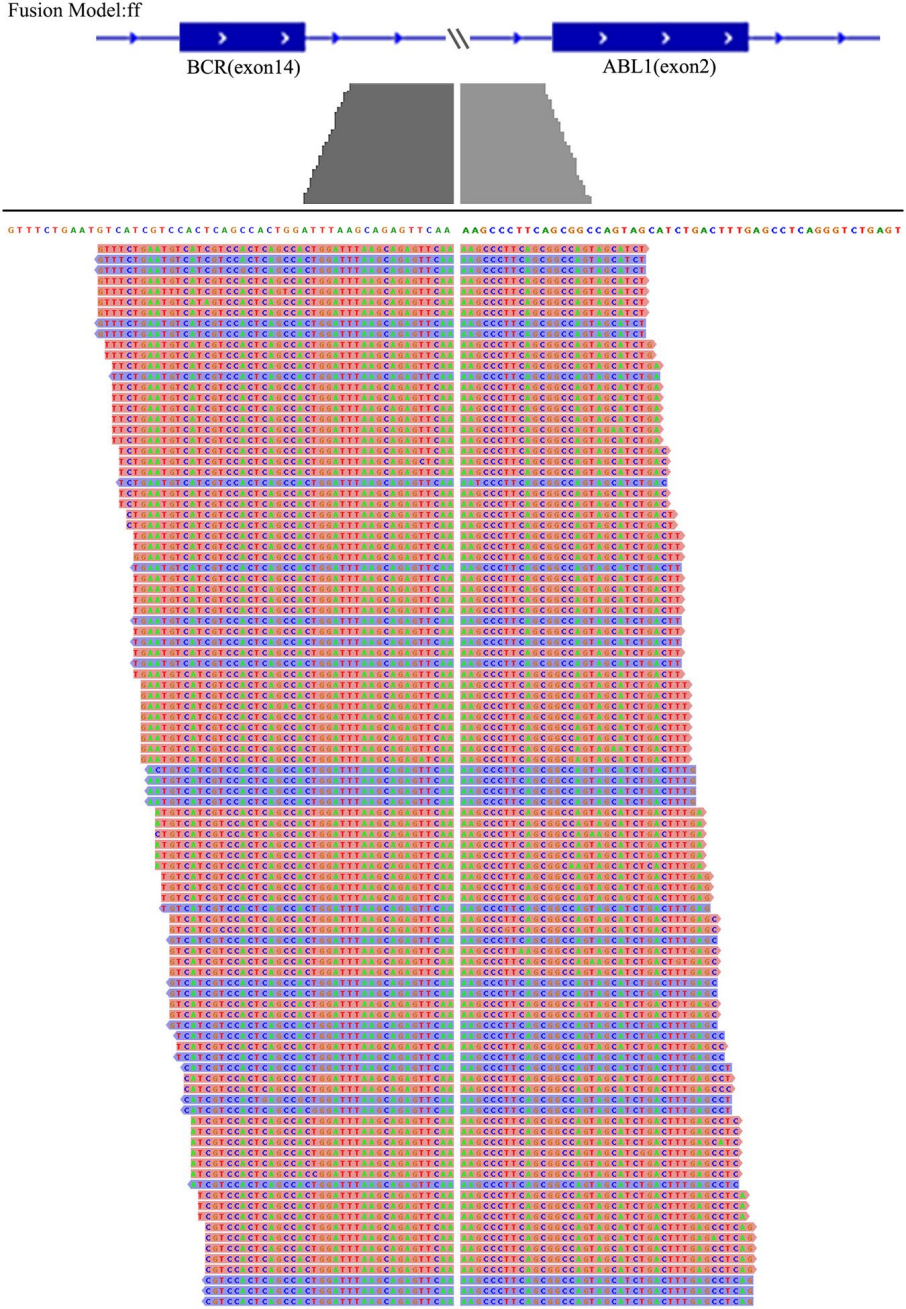
Split reads supporting BCR-ABL1 for K562 cell line single-end RNA-seq data.

We further applied GFusion on the published K562 CML cell line RNA-seq data containing 14 million 76 bp single-end reads. Two main high expression level gene fusions, BCR-ABL1 and NUP214-XKR3 already validated before were detected by GFusion^19, 36^. In the result, 281 split reads were found to bridge the fusion boundary of the fusion from upstream gene BCR at chr22:23632600 to downstream gene ABL1 at chr9:133729451 at the transcription level. As showed in Fig. 6, split reads are abundant for the fusion boundary between the chromosome 22 and 9, illustrating the fusion boundary precision achieved by GFusion even on single-end datasets.

In close proximity to BCR and ABL1, another gene fusion between NUP214 and XKR3 resided on chromosome 22 and 9 respectively was also detected. NUP214 have been known to be related in T-cell acute lymphoblastic leukemia^38^. One isoform of fusion NUP214 and XKR3 was identified by GFusion to be supported by 60 split reads connecting the end of exon 29 in NUP214 (chr9: 134074402) to the start of exon 2 in XKR3 (chr22:17288973). Another two additional isoform of fusion NUP214 and XKR3: exon 29 of NUP214 fused to exon 3 of XKR3, and exon 29 of NUP214 fused to exon 4 of XKR3 only supported by 2 and 8 split reads separately, were also detected by GFusion. The anchors orientation of the split reads detected by GFusion shows that fusion strand is forward to reverse. The fourth isoform of fusion NUP214-XKR3 involving exon 27 of NUP214 and exon 2 of XKR3 could also be found when we reduced the anchor length in GFusion. All of these detected fusion isoforms are in agreement with previous results^36^. In addition to BCR-ABL1 and NUP214-XKR3, GFusion identified all the other previously reported fusion genes, SNHG3-PICALM, PRIM1-NACA, NCKIPSD-CELSR3, SLC29A1-HSP90AB1. Among these six fusion genes, the two genes of fusion PRIM1-NACA, NCKIPSD-CELSR3, and SLC29A1-HSP90AB1 are close neighbors on the genome separately, and are likely to be fusion transcripts caused by read-through events^39^. The inner distances between two fused points on the genome are respectively 19 Kb in PRIM1-NACA, 21 Kb in NCKIPSD-CELSR3 and 15 Kb in SLC29A1-HSP90AB1.

To compare the performance of GFusion with FusionMap and Tophat-Fusion, we comprehensively analyzed their sensitivity, false positive rate as well as running time on the same dataset. As described in above results, GFusion detected the six fusion genes that are all previously reported by Levin *et al*.^36^ and predicted other 28 novel fusions (Table S3). Tophat-Fusion, by comparison, reported just three known fusion genes and 71 novel fusion genes with the parameters: fusion-min-dist = 10000, num-fusion-reads = 4, and missed three fusion PRIM1-NACA, NCKIPSD-CELSR3, and SLC29A1-HSP90AB1. Tophat-Fusion appears not to consider those read-through transcripts to be gene fusions. FusionMap detected five known fusions and predicted 221 novel fusion genes with the default parameters except: α = 25, β = 1, G = 2, MinimalHit = 2, MinimalFusionSpan = 10000 (Table S4)^22^. In addition, using the same computer as above-mentioned, GFusion took an hour that was five times faster than TopHat-Fusion. FusionMap is the fastest one which took only 10 minutes. Obviously, the problem concern of FusionMap is its sensitivity and lower false positive rate. GFusion is a superior and powerful tool for fusion gene detection using single-end reads that it shows greater sensitivity and has lower false discovery rate than Tophat-Fusion and FusionMap.

### Testing on Simulated Dataset

To further compare the performance of GFusion with the existing methods, we generated a simulation dataset that consists of a normal background data and artificial fusions. We used paired-end RNA-Seq data of human embryonic stem cells as the normal background dataset in which it was not expected to harbor any fusions. The background data used in our work can be downloaded from NCBI Sequence Read Archive (SRA: SRR521477) with approximately 24 million 76 bp paired-end reads generated by WiCell Research Institute. We firstly bridged sequences from exons of two different genes and obtained 42 artificial fusion transcripts. Secondly, the fusion supporting reads were generated at random to represent the expression level of artificial gene fusions and their insert segment length distribution was similar to the normal paired-end reads. Finally, the simulate dataset was produced by mixing the fusion supporting reads with normal background reads.

**Figure 7:**
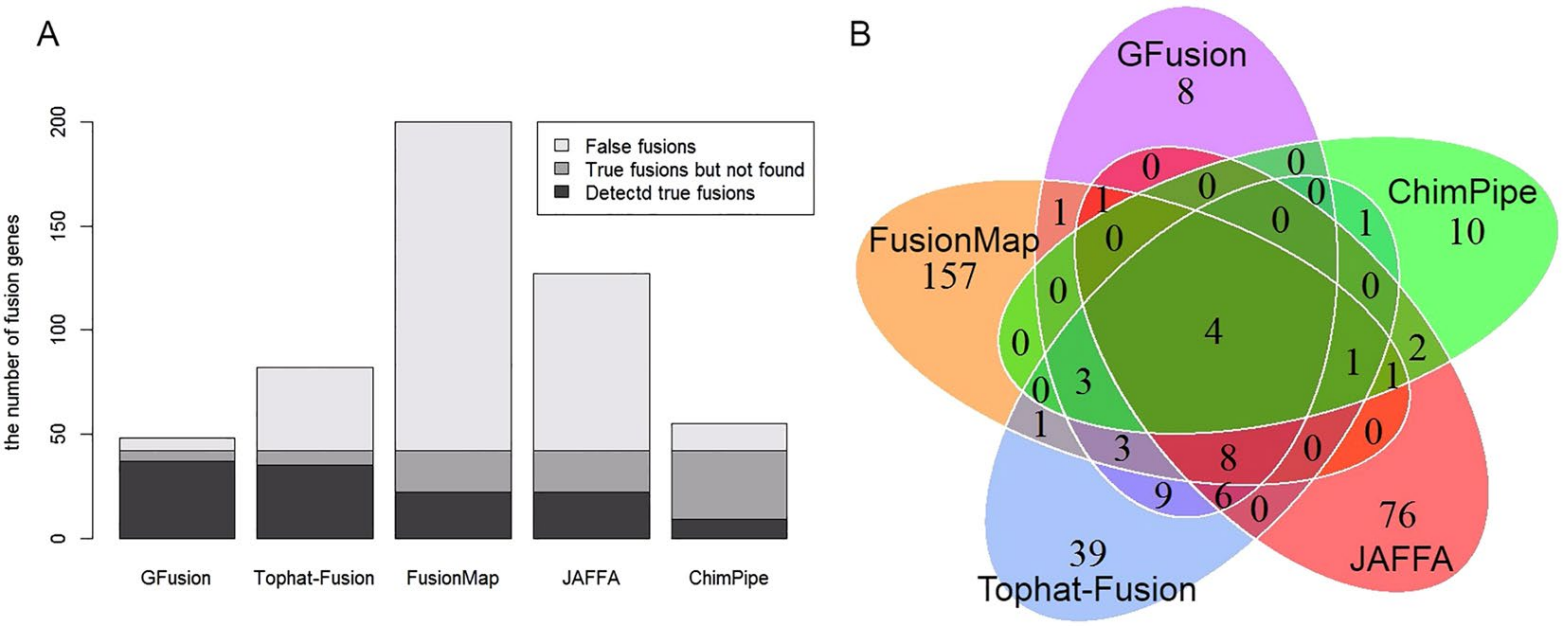
Comparison between GFusion, Tophat -fusion, FusionMap, JAFFA, and ChimPipe. (**A**) The bottom box of stacked bar shows the number of true fusion genes identified by tools, the middle box of stacked bar represent the number of the true fusions not detected by tools, the top box of stacked bar represent the number of false fusions. (**B**) Venn diagram comparison for the reported fusions by five tools.

After that, we ran GFusion, Tophat-Fusion, FusionMap, JAFFA, and ChimPipe on the simulated dataset, and counted the number of true and false supporting fusion reads to calculate sensitivity and false positive rate of three methods separately. It can be seen from Table 2 and Fig. 7A that GFusion had higher sensitivity and lower false positive rate than other four tools in the fusion detection on the simulated dataset. GFusion achieved a high sensitivity of 88% by identifying 37 out of 42 true fusion genes as well as just predicting seven false fusion genes with parameters: anchor length = 30, max intron length = 5000. Tophat-Fusion found 35 fusion genes with 83% sensitivity and reported 50 false fusion genes with parameters (fusion-min-dist = 5000, num-fusion-reads = 1). FusionMap reported 22 of 42 true fusion genes and 160 false fusions with parameters α = 30, β = 1, G = 2, MinimalHit = 1, MinimalFusionSpan = 5000. JAFFA detected 22 true fusion genes and 77 false fusions with the direct mode, and the hybrid mode was not used because this mode is very time-consuming. ChimPipe has the lowest sensitivity of 21% with the default parameters.

**Table 2:** Comparison of GFusion, Tophat-fusion, FusionMap, JAFFA, and ChimPipe on simulation dataset.

	True fusions	Sensitivity	False fusions	False positive rate(10^−5^)
GFusion	37	0.88	6	0.77
Tophat-fusion	35	0.83	40	3.95
FusionMap	22	0.524	158	2.37
JAFFA	22	0.524	77	–
ChimPipe	9	0.21	13	1.71

As shown in Table 2, the number of false positives detected by FusionMap were heavily more than that detected by other tools, especially comparing with GFusion. In addition, only 4 fusions were detected in common by all of the five methods as shown in Fig. 7B, and 12 fusions were detected at least four tools. 8 fusions were only found by GFusion, while only 1 fusions was found by other four tools but GFusion. This result also shows that these five tools are really different from each other in the detection of fusion genes.

For fusion detection, the number of supporting reads is another important indicator to confirm the fusions from candidate fusion genes. We calculated the Positive Predictive Values (PPV) with the number of supporting reads to estimate the fusion genes detection ability for these three pipelines.

2 $${\rm{PPV}}=\frac{TP}{TP+FP}\,$$

where TP means the number of true fusions; FP means the number of false fusion gene.

**Figure 8:**
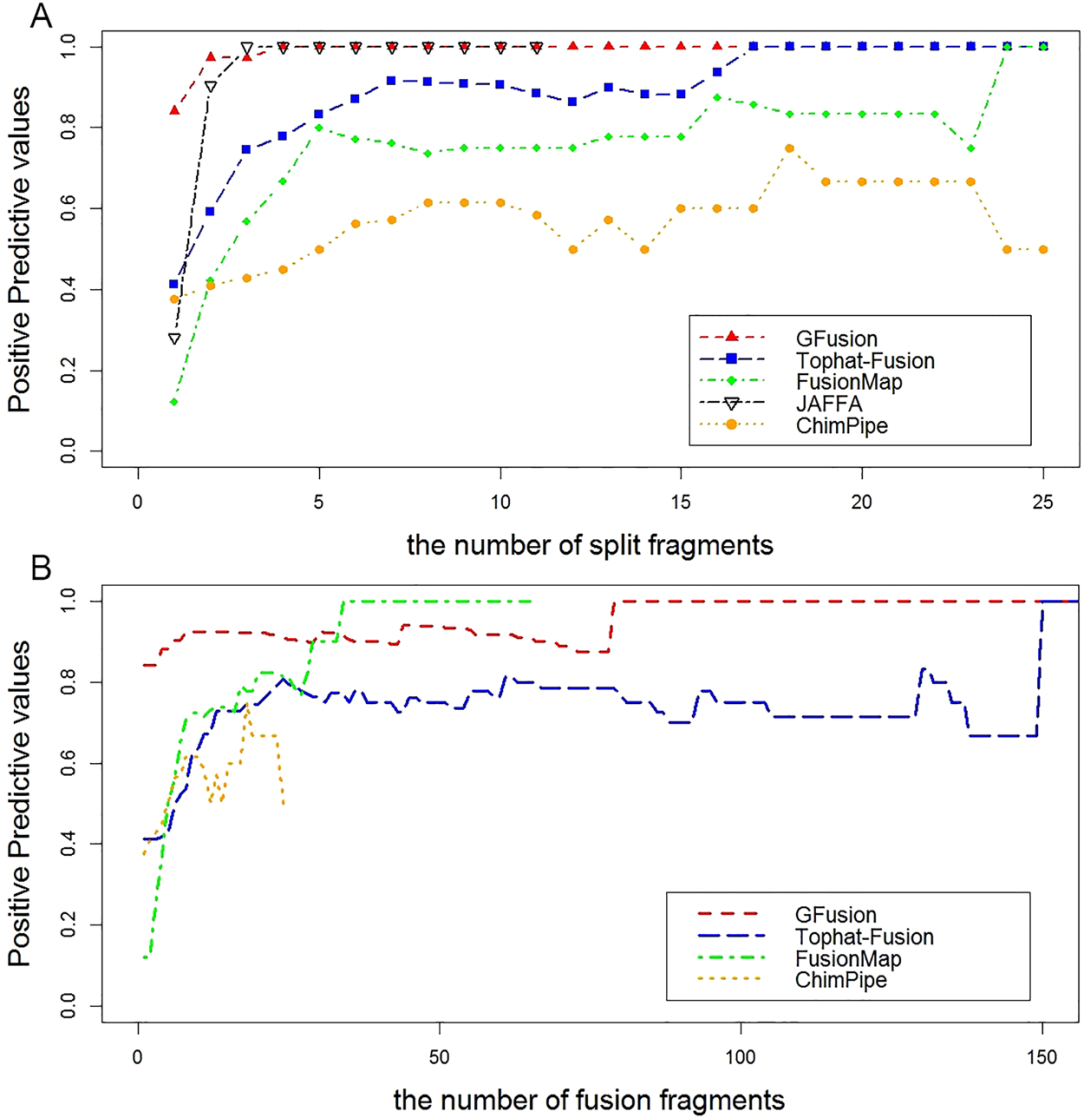
Positive Predictive Value varies with the number of supporting fragment. (**A**) PPV varies with split fragments. (**B**) PPV varies with all the number of fusion fragments (split fragments and spanning fragments).

GFusion reported the most number of true fusions and the fewest false fusions when supporting fragments number is greater than one. Considering the split fragments containing the fusion boundary in the reads (seed reads of FusionMap, spanning reads of Tophat-Fusion and JAFFA, nbSpanningReads of ChimPipe) (Fig. 8A), GFusion and JAFFA had higher PPV than other three tools. In addition, if we selected three split fragments as a minimum threshold to support the fusions, GFusion and JAFFA would filter out all the false fusion genes successfully. In contrast, Topaht-fusion, FussionMap and ChimPipe still predicted many false fusions. Furthermore, Tophat-fusion and ChimPipe could not filter out all the false fusion genes until the minimum supporting split fragments is set to be 17. By analyzing all the supporting split fragments and spanning fragments (rescued reads denoted in FusionMap, spanning pairs denoted in Tophat-Fusion, nbTotal denoted in ChimPipe) (Fig. 8B), the fusions reported by GFusion and Tophat-Fusion have more supporting fragments than FusionMap and ChimPipe. In addition, compared with Tophat-Fusion, GFusion has a higher PPV value which always kept stable greater than 80% as the supporting fragments increase, indicating that GFusion are more reliable in performance than Tophat-Fusion and FusionMap.

**Figure 9:**
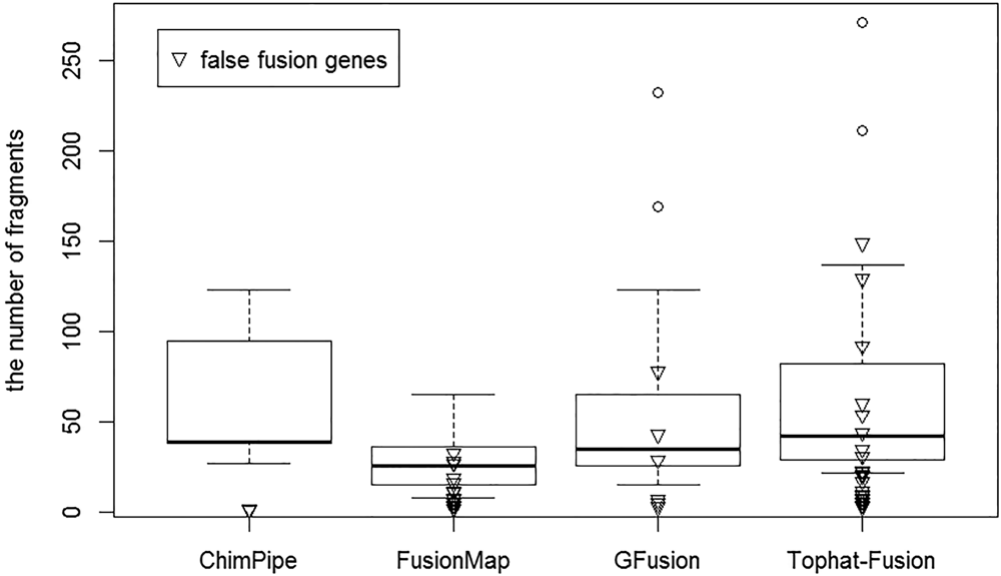
The distribution of supporting fragments for real or false fusion genes.

The distribution of supporting fragments for fusion genes can also be seen in Fig. 9, the number of false fusions supporting fragments reported by GFusion is relatively lower than the true fusions, as well as the ChimPipe. However in Tophat-fusion and FusionMap, the distribution of supporting fragments for true fusions is similar to the false fusions, and it is hard for these methods to reduce the false positive rate by setting the threshold of supporting fragments number.

## Conclusions

GFusion is an effective and reliable Perl script tool for fusion gene detection on both single-end and paired-end RNA-Seq data. GFusion can achieve highly sensitive and fewer false positive rate using split-realign protocols: splitting reads, realigning anchors, locating fusion boundaries and constructing fusion transcript reference. In order to remove the false fusion genes and keep the higher sensitivity, GFusion designed several strict filtering steps including locating the reads position, confirming transcribing orientations, constructing fusion reference, and realigning candidate fusion fragments. Compared with discordant pairs in which each reads align against different genes or individual unmapped reads harboring the fusion boundaries, GFusion focuses more on the mapped mate reads and split reads. GFusion requires the anchor length more than 20 bp to minimize impact of short sequence multi-mapping.

We have tested the performance of the GFusion on published RNA-Seq datasets with known fusion genes involving paired-end and single-end read dataset as well as a simulated dataset. Both of these results indicated that GFusion performed higher sensitivity and far lower false positive rate compared with other existing fusion detection methods.

## Electronic supplementary material

Supplementary File
