# Supplementary material for: GFusion: an Effective Algorithm to Identify Fusion Genes from Cancer RNA-Seq Data
Source: Sci Rep. 2017 Jul 31;7:6880. doi: 10.1038/s41598-017-07070-6 (PMC5537242; doi:10.1038/s41598-017-07070-6)
Supplement: Supplementary file 1 — Supplementary File [file 41598_2017_7070_MOESM1_ESM.pdf]

GFusion: an Effective Algorithm to Identify Fusion Genes from Cancer RNA-Seq Data

Jian Zhao[1], Qi Chen[1], Jing Wu[1], Ping Han[2,*], Xiaofeng Song[1,*]

1 Department of Biomedical Engineering, Nanjing University of Aeronautics and Astronautics, Nanjing 210016, China;

2 Department of Gynecology and Obstetrics, The First Affiliated Hospital with Nanjing Medical University, Nanjing 210029, China;

*Co-corresponding authors: xfsong@nuaa.edu.cn; hanping200701@163.com

Table S1 The definition of four fusion models

Fusion Model	Description
ff	3’-end of the upstream gene combines with 5’-end of the downstream genes in the sense strand.
fr	3’-end of the upstream gene in the sense strand combines with 5’-end of the downstream genes in the antisense strand.
rr	3’-end of the upstream gene combines with 5’-end of the downstream genes in the antisense strand.
rf	3’-end of the upstream gene in the antisense strand combines with 5’-end of the downstream genes in the sense strand.

Table S2 GFusion predicts for breast cancer and Normal breast cell lines

**Sample**	**Fusion genes (5’-3’)**	**Chromosom: Position (5’)**	**Chromosom: Position (3’)**	**Strand (5’-3’)**	**Split reads**	**Spanning reads**
**MCF7**	**BCAS4-BCAS3**	**chr20:49411710**	**chr17:59445688**	**ff**	**45**	**103**
**MCF7**	**ARFGEF2-SULF2**	**chr20:47538547**	**chr20:46365686**	**fr**	**20**	**5**
**MCF7**	**RPS6KB1-VMP1**	**chr17:57992064**	**chr17:57917129**	**ff**	**3**	**1**
**MCF7**	**SMARCA4-CARM1**	**chr19:11097269**	**chr19:11015627**	**ff**	**3**	**2**
MCF7	MYO9B-FCHO1	chr19:17213367	chr19:17881234	ff	2	1
MCF7	PAPOLA-AK7	chr14:96968937	chr14:96904172	ff	2	1
**SK-BR-3**	**TATDN1-GSDMB**	**chr8:125551266**	**chr17:38063240**	**rr**	**1**	**9**
**SK-BR-3**	**TATDN1-GSDMB**	**chr8:125551266**	**chr17:38066177**	**rr**	**142**	**9**
**SK-BR-3**	**TATDN1-GSDMB**	**chr8:125551266**	**chr17:38062524**	**rr**	**24**	**9**
**SK-BR-3**	**TATDN1-GSDMB**	**chr8:125551266**	**chr17:38062238**	**rr**	**33**	**9**
**SK-BR-3**	**RARA-PKIA**	**chr17:38465538**	**chr8:79510593**	**ff**	**1**	**12**
**SK-BR-3**	**RARA-PKIA**	**chr17:38465538**	**chr8:79485046**	**ff**	**5**	**12**
**SK-BR-3**	**ANKHD1-PCDH1**	**chr5:139825560**	**chr5:141234001**	**fr**	**5**	**7**
**SK-BR-3**	**CCDC85C-SETD3**	**chr14:100002352**	**chr14:99880271**	**ff**	**4**	**3**
**SK-BR-3**	**SUMF1-LRRFIP2**	**chr3: 4494560**	**chr3:37170640**	**rr**	**1**	**5**
**SK-BR-3**	**SUMF1-LRRFIP2**	**chr3:4418014**	**chr3:37170640**	**rr**	**3**	**5**
**SK-BR-3**	**WDR67- ZNF704**	**chr8:124096580**	**chr8:81733850**	**fr**	**2**	**3**
**SK-BR-3**	**CYTH1-EIF3H**	**chr17:76778284**	**chr8:117738411**	**rr**	**1**	**19**
**SK-BR-3**	**CYTH1-EIF3H**	**chr17:76778284**	**chr8:117671219**	**rr**	**1**	**19**
SK-BR-3	EMB-EMBP1	chr5:49723978	chr1:121298308	rf	1	1
SK-BR-3	ALDH3B1-ALDH3B2	chr11:67790229	chr11:67433142	fr	1	35
SK-BR-3	ALDH3B1-ALDH3B2	chr11:67790229	chr11:67430870	fr	1	35
SK-BR-3	P2RY6-ARHGEF17	chr11:72983511	chr11:73057928	ff	1	1
**BT-474**	**ACACA-STAC2**	**chr17:35479453**	**chr17:37374426**	**rr**	**33**	**43**
**BT-474**	**RPS6KB1-SNF8**	**chr17:47021337**	**chr17: 57970686**	**fr**	**22**	**25**
**BT-474**	**RPS6KB1-SNF8**	**chr17:47021337**	**chr17: 57971285**	**fr**	**1**	**25**

**BT-474**	**VAPB-IKZF3**	**chr17:37934020**	**chr20:56964573**	**fr**	**3**	**35**
**BT-474**	**VAPB-IKZF3**	**chr17:37922746**	**chr20:56964573**	**fr**	**9**	**35**
**BT-474**	**ZMYND8-CEP250**	**chr20:45852970**	**chr20:34078463**	**rf**	**4**	**33**
**BT-474**	**RAB22A-MYO9B**	**chr20:56886178**	**chr19:17256207**	**ff**	**3**	**6**
**BT-474**	**TRIM37-MYO19**	**chr17:34863763**	**chr17:57161363**	**rr**	**1**	**1**
**BT-474**	**DIDO1-TTI1**	**chr20:61569148**	**chr20: 36634799**	**rr**	**2**	**4**
**BT-474**	**STARD3-DOK5**	**chr17:37793484**	**chr20:53259997**	**ff**	**2**	**6**
**BT-474**	**GLB1-CMTM7**	**chr3:33055548**	**chr3:32483332**	**rf**	**1**	**5**
**BT-474**	**GLB1-CMTM7**	**chr3:33055548**	**chr3:32490946**	**rf**	**1**	**5**
**BT-474**	**CPNE1-PI3**	**chr20:34243124**	**chr20:43804502**	**rf**	**1**	**3**
**BT-474**	**MED1-ACSF2**	**chr17:37595418**	**chr17:48548389**	**rf**	**6**	**9**
**BT-474**	**MED1-STXBP4**	**chr17:37595418**	**chr17:53218671**	**rf**	**7**	**1**
**BT-474**	**STX16-RAE1**	**chr20:57227143**	**chr20:55929088**	**ff**	**3**	**19**
**BT-474**	**TOB1-SYNRG**	**chr17:48943419**	**chr17:35880751**	**rr**	**7**	**21**
**BT-474**	**MED13- BCAS3**	**chr17:60129898**	**chr17:59469338**	**rf**	**2**	**2**
**BT-474**	**TRPC4AP-MRPL45**	**chr20:33665849**	**chr17:36478009**	**rf**	**1**	**3**
**BT-474**	**TRPC4AP-MRPL45**	**chr20:33665849**	**chr17:36476502**	**rf**	**3**	**3**
**BT-474**	**PIP4K2B-RAD51C**	**chr17:36933940**	**chr17:56811479**	**rf**	**1**	**1**
**BT-474**	**PIP4K2B-RAD51C**	**chr17:36933940**	**chr17:56809845**	**rf**	**1**	**1**
BT-474	FITM2-UQCC	chr20:42939616	chr20:33902568	rr	2	1
Normal	SLC2A14-SLC2A3	chr12:7973817	chr128077107	rr	1	1

20 known fusion genes in breast cancer with boldface and 15 novel fusions in four breast cell line.

Table S3 GFusion predicts for k-562 cell lines

Sample	Fusion genes (5’-3’)	Chromosom: Position (5’)	Chromosom: Position (3’)	Strand (5’-3’)	Split reads
**K-562**	**BCR-ABL1**	**chr22:23632600**	**chr9:133729451**	**ff**	**281**
**K-562**	**NUP214-XKR3**	**chr9:134074402**	**chr22:17265299**	**fr**	**8**
**K-562**	**NUP214-XKR3**	**chr9:134074402**	**chr22:17280914**	**fr**	**2**
**K-562**	**NUP214-XKR3**	**chr9:134074402**	**chr22:17288973**	**fr**	**60**
**K-562**	**SNHG3-PICALM**	**chr1:28832596**	**chr11:85695016**	**fr**	**2**
**K-562**	**SNHG3-PICALM**	**chr1:28834672**	**chr11:85695016**	**fr**	**1**
**K-562**	**PRIM1-NACA**	**chr12:57127931**	**chr12:57108471**	**rr**	**1**
**K-562**	**NCKIPSD-CELSR3**	**chr3:48715997**	**chr3:48694781**	**rr**	**4**
**K-562**	**SLC29A1-HSP90AB1**	**chr6:44200165**	**chr6:44216367**	**ff**	**3**
K-562	ACCS-EXT2	chr11:44089465	chr11:44129233	ff	2
K-562	ACCS-EXT2	chr11:44097142	chr11:44129233	ff	1
K-562	ACCS-EXT2	chr11:44104861	chr11:44129233	ff	1
K-562	BRK1-VHL	chr3:10157503	chr3:10191471	ff	1
K-562	BCR-ZNF138	chr22:23632600	chr7:64291318	ff	2
K-562	CLN6-ABL1	chr15:68510874	chr9:133729451	rf	1
K-562	EIF4A2-VPS36	chr3:186505373	chr13:52990228	fr	1
K-562	FYN-YES1	chr6:112015578	chr18:732965	rr	4
K-562	GNAS-GNAI3	chr20:57464408	chr1:110116359	ff	2
K-562	LCOR-GNAS	chr10:98592156	chr20:57470667	ff	3
K-562	MSI1-MSI2	chr12:120800846	chr17:55607037	rf	2
K-562	MYH14-MYH9	chr19:50728934	chr22:36717866	fr	3
K-562	MYO1D-BRIP1	chr17:31065271	chr17:59878835	rr	2
K-562	NACA-PCBP2	chr12:57107321	chr12:53845886	rf	2
K-562	NELFA-WHSC1	chr4:2010477	chr4:1932353	rf	1
K-562	NKTR-CLTC	chr3:42674315	chr17:57754315	ff	1
K-562	NR3C2-NR3C1	chr4:149181130	chr5:142689778	rr	4
K-562	PI4KA-CRKL	chr22:21212858	chr22:21303999	rf	1
K-562	PRKCE-PRKCB	chr2:46234800	chr16:24166005	ff	1
K-562	SBDS-SBDSP1	chr7:66460277	chr7:72301272	rf	8
K-562	SNHG3-CARS	chr1:28832596	chr11:3033506	fr	2
K-562	SNHG3-PCM1	chr1:28832596	chr8:17868746	ff	2
K-562	SSX8-SSX1	chrX:52654666	chrX:48117971	ff	1
K-562	SSX8-SSX1	chrX:52655437	chrX:48121201	ff	1
K-562	TAF15-FUS	chr17:34165557	chr16:31201361	ff	1
K-562	TBC1D3-USP6	chr17:36288301	chr17:5037182	ff	1
K-562	TFEB-TFE3	chr6:41655491	chrX:48891055	rr	3
K-562	TMA16-GNAS	chr4:164415956	chr20:57470667	ff	1
K-562	TNRC6A-AAK1	chr16:24809287	chr2:69771676	fr	3
K-562	TUBA3FP-CRKL	chr22:21368071	chr22:21288067	rf	1
K-562	ZNF169-GNAS	chr9:97055351	chr20:57470667	ff	1

The 34 candidate fusion genes found by GFusion in K-562 cell line, containing six previously known fusions and

28 novel fusions.

Table S4 The parameters of FusionMap for fusion detection on K-562 cell line FusionMap was run for K-562 cell line using Genome reference library and gene model for Human.B37.3 that could download from http://omicsoft.com/downloads/dreflib/ .The parameters of FusionMap was set as follows:

**parameters**	**value**
FilterUnlikelyFusionReads	False
FullLengthPenaltyProportion	8
MinimalFusionAlignmentLength	25
FusionReportCutoff	1
NonCanonicalSpliceJunctionPenalty	2
MinimalHit	2
MinimalRescuedReadNumber	1
MinimalFusionSpan	1
RealignToGenome	True
OutputFusionReads	True
Default value	True
